# Induction of lutein production in *Scenedesmus obliquus* under different culture conditions prior to its semipreparative isolation

**DOI:** 10.55730/1300-0527.3369

**Published:** 2022-02-03

**Authors:** Ayşegül ERDOĞAN, Ayça Büşra KARATAŞ, Zeliha DEMİREL, Meltem CONK DALAY

**Affiliations:** 1 Ege University Application and Research Centre For Testing and Analysis (EGE MATAL), İzmir, Turkey; 2 Department of Bioengineering, Faculty of Engineering, Ege University, İzmir, Turkey

**Keywords:** Lutein, green microalga, extraction, purification, preparative chromatography

## Abstract

Microalgae with their improved growth rates and accumulation of high-value-added products make their commercial production attractive. Among them, lutein, which is a carotenoid, plays a very important role due to its various applications in the food and pharmaceutical industry. Induction of its biosynthesis can be triggered by various stress conditions like light. In this study, three different light intensities (50,150 and 300 μmol photons/m^2^s) and aeration rates (1, 3, and 5 L/min) were utilized to induce the lutein biosynthesis and biomass productivity in *Scenedesmus obliquus*. Lutein was isolated by preparative chromatography using a semiprep C_30_ column (10 × 250 mm, 5μm) and its confirmation was made by LC-MS/MS. According to the results, *Scenedesmus obliquus* synthesized the maximum lutein (8.01 ± 0.1 mg/g) with biomass productivity of 1.698 g/L at 150 μmol photons/m^2^s light intensity using 3 L/min as aeration rate. To the best of the authors’ knowledge, this was the first study that the lutein was isolated by preparative chromatography using semiprep C_30_ carotenoid column with a simple and rapid separation, which can be used as a reference methodology for the isolation of other carotenoids. *Scenedesmus obliquus* can be an important alternative source for commercial production of lutein, as it is indicated from the results of this study.

## 1. Introduction

Today, microalgae are used in aquaculture, food sector and cosmetics industry for various purposes. In addition, microalgae are preferred in the production of different substances because they are rich in high-value components such as polyunsaturated fatty acids and carotenoids [1]. Carotenoids, which are isoprenoid compounds with the C_40_ structure, are found in a variety of organisms such as bacteria, archaea, fungi, algae, plants and animals. More than 750 natural carotenoids have been isolated and identified to date [2]. Carotenoids are valuable components of the photosynthetic machine in photoautotrophs. They play an important role in collecting light to help remove reactive oxygen species by providing photosynthesis and thermal dispersion and protect photosynthesis II (PSII) from photodamage [3–5]. The environmental stimuli can also alter the biosynthesis mechanism and the amount of photosynthetic pigments [6,7]. Microalgae have several light-collecting carotenoid pigment molecules such as lutein, zeaxanthin, canthaxanthin, astaxanthin, and β-carotene. Lutein is one of the important xanthophylls found in plants such as spinach, cabbage, and marigold, as well as some green microalgae species such as *Scenedesmus almeriensis, Muriellopsis* sp. and *Chlorella zofingiensis* [8]. It is a fat-soluble carotenoid that people get from their diets and has many beneficial health effects, such as helping prevent macular degenerative disease, reducing the risk of heart attack and alleviating against other metabolic syndromes. The reported benefits of lutein, a natural food colour and potentially a high-value nutraceutical functional food ingredient, have led to more research and studies on it [9].

Primarily, the commercial production of natural lutein relies on obtaining it from marigold flower [10]. On the other hand, marigold has been harvested and extracted seasonally and requires intensive effort. Studies have suggested that microalgal species under specified growing conditions with tedious control can produce much higher lutein than marigold varieties [11]. Several microalgae are potential sources of lutein as they have high lutein content (0.5%–1.2% dry weight) [12–14]. Therefore, these organisms have the potential to be alternatives for lutein production. Microalgae can be grown all year round, depending on the photobioreactor growing conditions chosen. In addition, they also have high biomass productivity and lutein content. Several microalgae species are potential sources of lutein as they produce about 5 g/kg biomass mainly in free lutein form [15]. For this reason, lutein obtained from microalgae is an important candidate in terms of industrial production. Additionally, there are many ways to increase the lutein content of microalgae, such as different light intensities, temperature, and some nutritional restrictions. Ho, Chan [16] applied some strategies using light to enhance cell growth and lutein amount (4.61 mg/g) of *Scenedesmus obliquus* FSP-3, achieving the best lutein productivity (4.08 mg/Ld) at a light intensity of 300 μmol photons/m^2^s. In another study, Zhao et al. [17] worked under different environmental conditions for the production of lutein and concluded that the optimum lutein content was achieved under a lower temperature of 20–25 °C using blue light. Schüler et al. [18] also studied with marine microalgae *Tetraselmis sp*. CTP4 for carotenoid biosynthesis in which the amount of lutein increases 1.5 times at higher light intensities (170 and 280 μmol photons/m^2^), but they ​​found a temperature increase of 20 to 35 °C after just two days at 170 μmol photons/m^2^s light intensity and achieved the highest lutein productivity (3.17 mg/g dry weight) under these conditions. Additionally, another research also described light stress on marine microalgae *Chlamydomonas* sp. JSC4 as a potential lutein producer [19]. They achieved high lutein amount (5.08 mg/Ld) under high light of 625 μmol photon/m^2^s, and the amount of lutein decreased at 750 μmol photons/m^2^s due to down-regulation of lut1 and zep gene. They encoded the ɛ-carotene hydroxylase and zeaxanthin epoxidase enzymes responsible for lutein biosynthesis, respectively. Chen et al. [20] also introduced carotenogenesis strategies based on light with different nitrogen concentrations and growth conditions on *Scenedesmus obliquus* CWL. In contrast to Ho et al. [16], they obtained a maximum lutein yield (1.43 mg/Ld) under blue/red light. There is currently no commercial production system for lutein from microalgae; but *Muriellopsis* sp. and *Scenedesmus* sp. pilot scale trials were conducted. *Muriellopsis sp*. was grown in a 55 L tubular photobioreactor and the highest lutein productivity was reported as 40 g/m^2^d. *Scenedesmus almeriensis* was cultivated for lutein production in a 4000 L tubular photobioreactor and it was observed to produce 290 g/m^2^d lutein [21]. Although marigold meets global lutein demand to some extent, natural lutein production is still needed to meet global demand. In this sense, microalgae play an important role in this regard as there are many microalgae that produce lutein at 0.5%–1.2% of dry cell weight [11]. Also, there is more free lutein in microalgae than in marigold. Additionally, advanced metabolic engineering strategies can be applied to increase the amount of lutein. Overall, given the increasing demand for natural lutein, the current potential type of microalgae, and the growing technologies available, some research activities for microalgal lutein production and industrial production need to be accelerated. The main approach here would be to rapidly grow selected microalgae species by altering abiotic growth factors to increase lutein content, followed by simple collection of biomasses, cost-effective lutein extraction, and evaluation of biomass extracted from lutein for additional bioproducts. For these reasons, information is still needed on optimizing the culture conditions of lutein-producing microalgae to improve content. In this study, lutein biosynthesis, and accumulation and aeration rate were investigated in *Scenedesmus obliquus*, a green microalga in response to light. Then, the enhanced amount of lutein was purified by preparative chromatography using a semiprep C_30_ column. Finally, the isolated lutein fraction was confirmed by mass spectrometry. With this study, lutein biosynthesis in *S. obliquus* achieved maximum efficiency (8.01 mg/g) under 150 μmol photon/m^2^s light intensity with a aeration rate of 3 L/min.

## 2. Materials and methods

### 2.1. Reagents and chemicals

All-trans-lutein, triethylamine, pyrogallol, and calcium carbonate were provided by Sigma-Aldrich (St Louis, MO, USA), and all the solvents used in this study were LC-grade purchased from Merck.

### 2.2. Growth conditions and harvesting of *S*. *obliquus*

Green microalga was provided by the Ege University Microalgae Culture Collection EGEMACC (htps://egemacc.com/cultures.php). The species of green microalgae and culture collection codes was given as *S. obliquus* EGEMACC-18. It was cultivated in BBM medium [22]. The inoculum was added in a bubble-column photobioreactor (2 L) and cultivated under the light intensity of 50, 150 and 300 μmol photons/m^2^s (LED-Cata10W CT-5254), at 21 ± 2 °C, and with the aeration rate of 1, 3 and 5 L/min. The growth of microalgae was pursued by counting microalgae cells using Neubauer counting chamber and measuring optical density at 680 nm at 2-day intervals over the period of 16 days.

The specific growth rate for *S. obliquus* was calculated according to Becker [23] (Eq. 1) using the data obtained by the absorbance values taken at 680 nm.


$$\mu =\frac{lnx2-\text{ln }x1}{\Delta t}$$

where μ= specific growth rate, x_2_ = cell concentration at time, t_2_, x_1_ = cell concentration at time t_1_, and Δt = t_2_-t_1_. Doubling time was determined as 0.693/μ.

Microalgal cells reached the stationary phase harvested by centrifugation at 6000 rpm and 4 °C for 7 min (ProResearch, By Centurion Scientific Ltd, UK). After, the pellet was washed with deionized water to remove the growing medium. Then, the wet biomass was lyophilized (Christ Alpha 1–2 LD plus, Germany) and grounded using a mortar for the extraction process. They were stored at −20 °C under dark conditions. Finally, the change in morphology of *S. obliquus* was also examined by scanning electron microscopy (SEM) after each growth condition was applied.

### 2.3. Ultrasound assisted extraction and saponification of lutein

For the extraction of lutein, a modified protocol was applied reported in our previous study [24]. For this purpose, 0.20 g of dry biomass was weighed and 0.20 g of CaCO_3_ was added. The mixture was extracted in an ultrasonic bath (Elmasonic S80H) using 10.0 mL of ethanol containing 0.01% pyrogallol for 10 min at 30 °C. Then, the solution was centrifuged for 2 min at 5000 rpm. The supernatant was kept, and the residue was reextracted with a fresh ethanol until decolouration of biomass. Finally, the combined solutions were filtered by vacuum filtration using 47 mm of 0.20 μm nylon filter paper (Sartorius). After, 10% of methanolic KOH was added to the extract for saponification. This process took 4 h to obtain the maximum lutein content (data not shown). In order to stop the saponification process, 10.0 mL of 10% Na_2_SO_4_ solution (w/v) was added. Then, 10.0 mL of diethyl ether was introduced to pool the carotenoid fraction form the extract. The yellow-orange upper phase was collected, and this was repeated until this phase was colourless. Then, some CaCl_2_ was added to the mixture to remove the residual water. Next, the carotenoid extract was filtered by nylon filter paper again and the solution was evaporated at 40 °C under 400 mbar by a rotary evaporator (Stuart RE 400). Finally, the residue was dissolved in 5.0 mL of chloroform stabilized with 1% ethanol and kept at −20 °C prior to HPLC analysis. All the experiments were performed in triplicate.

For the accurate determination of lutein in *S. obliquus*, method validation was performed using BCR certified Reference Material (Mixed vegetables, Merck BCR 485). All the procedures were applied in the same manner and the lutein content was determined.

### 2.4. Method validation

Certified reference materials (CRMs) play a critical role in validating the accuracy of compounds in a food matrix. In addition, care must be taken in choosing CRMs to monitor food composition analysis. As the BCR 485-Mixed Vegetables (Sigma-Aldrich, St. Louis, MO, USA) composition is closer to the green microalgae, it was chosen as the matrix to check the accuracy of the method for the determination of lutein. In the present study, lutein was found to be 12.1 ± 0.6 mg/kg after three repeated experiments.

### 2.5. Preparation of stock and standard lutein solutions

For the preparation of 100.0 mg/L stock lutein solution, 1.0 mg of lutein was weighed and dissolved in 10.0 mL chloroform (stabilized with 1 % ethanol).

Calibration standards were prepared by using this stock solution for the construction of a calibration curve. All standard solutions were kept in amber-coloured volumetric flasks. Different concentrations (0.01–10.0 mg/L) of lutein standards were injected into the HPLC-DAD, and the linear regression equation was acquired (y = 656.81x − 147.8; r^2^ = 0.9998).

### 2.6. Determination of microalgal lutein content

The lutein content in the carotenoid extract was determined by diluting it with mobile phase (Methanol: Methyl-tert-butyl ether: Water/70:25:5) before injecting 20 μL to the HPLC (1260 Series, Agilent, USA) with a diode array detector. The carotenoid extract was eluted at a flow rate of 1.0 mL/min at 25 °C at 446 nm (at a wavelength of maximum absorbance for lutein) using Waters YMC-C_30_ Carotenoid column (4.6 × 250 mm, 5μm). Gradient mobile phase system containing Methanol: Methyl-tert-butyl ether: Water was used according to the previous studies [24,25].

### 2.7. Isolation of lutein by prep-HPLC and instrumental conditions for LC-MS/MS

The preparative purification was performed using Thermo Fisher Scientific Ultimate 3000 HPLC system with a YMC-C_30_ semiprep Carotenoid column (10 × 250 mm, 5μm). The mobile phase system was a binary gradient system with at a flow rate of 4.0 mL/min at 446 nm with a column temperature of 25 °C and the injection volume was 2.0 mL. The gradient mobile phase was as followed: the collected fractions were then combined and separated into two before evaporation under nitrogen gas. Each was dissolved in methanol for further LC-MS/MS analysis.

In the present study, the isolated lutein obtained from S*. obliquus* extract was confirmed by liquid chromatography-tandem mass spectrometry (LC-MS/MS) equipped with an atmospheric pressure chemical ionization probe (APCI). The mass spectrometer (Thermo Scientific/TSQ Quantum Access Max) was operated in full scan mode from m/z 50–900 at 350 °C for vaporization temperature.

## 3. Results and discussion

### 3.1. Morphological changes in *S*. *obliquus*

**Figure 1:**
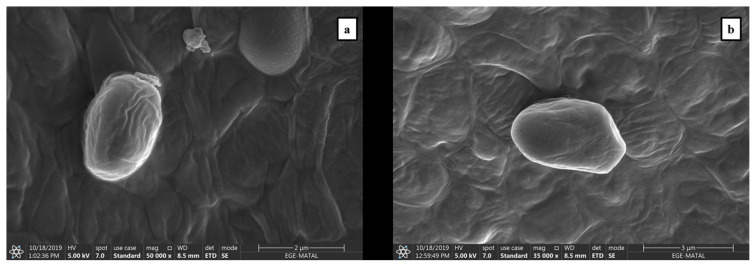
SEM images of *S. obliquus* cultivated under different culture conditions. a. S1-300: 300 μmol photons/m^2^s and 1 L/min aeration rate, b. 150 μmol photons/m^2^s and 3 L/min aeration rate.

To examine the morphological changes in *S. obliquus* in variation with light intensity and aeration rate, SEM images were taken for each species. Figure 1a belongs to cells in the light intensity of 300 μmol photons/m^2^s and 1 L/min aeration rate that produces lowest amount of lutein and Figure 1b presents the images of the cells in 150 μmol photons/m^2^s and 3 L/min (S3-150). When these images were checked, it was observed that there were changes in the dimensions of *S. obliquus*. Cell sizes increased approximately 1.5 (from 1.75 μm × 2.57 μm to 2.32 μm and 3.85 μm) times both transversely and longitudinally. This enlargement in the cell size could be supported with the increase in the lutein content. Probably, the cells are considered to require a larger area to keep the increased lipid and carotenoid amount.

### 3.2. Effect of aeration rate and light intensity on lutein content and biomass productivity

Synthesis of lutein, which is among the carotenoids with the strongest antioxidant properties [26–28], can be triggered excessively when exposed to high light. It is a very interesting pigment structurally because it is present in many photosynthetic cells.

Photosynthetic cells and their synthesis may increase under photooxidative stress. In general, with some exceptions, an increase in lutein pigment has been observed in the literature with increasing light intensity. One of these is the study of *Murellopsis* sp. When the light intensity of 460 μmol photons/m^2^s is applied, the amount of lutein reaches the highest level [29]. It was emphasized the lutein amount in *Scenedesmus almeriensis* also increases with the increase of the light intensity. In the study conducted by Sanchez et al. [30], *S. almeriensis* has been reported to produce high amounts of lutein using 1625 μmol photons/m^2^s light intensity at 35 °C. In this case, use of high light and high temperature gave a combined effect leading to an increase in lutein. Previous research conducted with *S. obliquus* FSP-3 has shown that a relatively high amount of lutein content (4.52 mg/g) and productivity under the light intensity of 300 μmol photons/m^2^s [16]. In another work, it was observed that the amount of lutein in *Desmodesmus sp*. also increased with a light intensity of 600 μmol photons/m^2^s [31]. Lutein content was reported to be 5.05 mg/g which is very high. Ma et al. [19] also examined light stress on the marine microalga *Chlamydomonas sp*. JSC4 as a potential lutein production. High lutein productivity (5.08 mg/Ld) was achieved under high light irradiation of 625 μmol photons/m^2^s.

In the present work, in order to increase the lutein content in *Scenedesmus obliquus* cells, three different light intensities (50, 150, 300 μmol photons/m^2^s) and different aeration rates (1, 3, 5 L/min) were used in 2 L photobioreactors. They were cultivated for 16 days. Two-day-old samples were taken and cell count, optical density, cellular increase and microalgal growth were determined. Lutein analysis was performed from the biomasses obtained at the end of the 16th day and the amount found in the cells produced in the experimental sets was determined. When different light intensities and aeration rates were tested, there was a decrease in the amount of lutein in *S. obliquus* and increase in cell number and growth rate. When appropriate aeration rate and light intensity is used, the amount of lutein has increased (Table 1).

**Figure 2:**
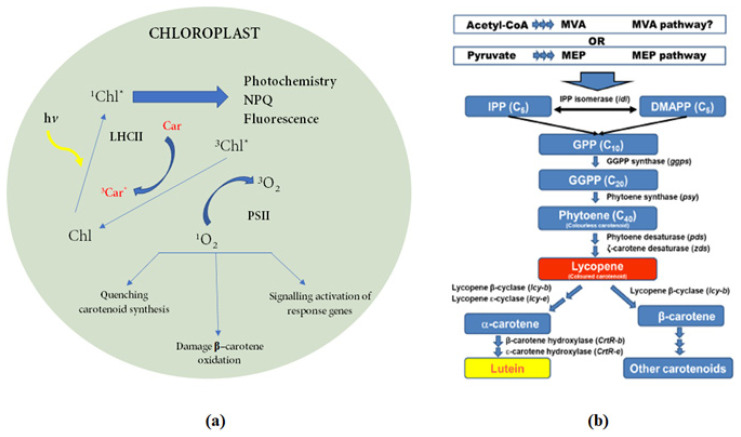
(a) Schematic description of the induction of photoprotection by triggering carotenoid synthesis via high light stress, (b) lutein synthesis in microalgae involving the corresponding genes and enzymes in each step [9, 33].

The amount of dry weight of *S. obliquus*, increased at 300 μmol photons/m^2^s, which is the highest light intensity, while the highest biomass (0.65 ± 0.07 g/L) was detected at 1 L/min aeration rate. At the same light intensity, at an aeration rate of 3 L/min, the doubling time and dry weight were found to be 2 days and 0.48 ± 0.08 g/L, respectively. Almost 15% reduction in the lutein amount under high illumination can be due to the decrease in the size of light harvesting antenna complex as the lutein is reported to localize here [32]. While biomass increase occurred in 1 L/min and 3 L/min aeration rates, the amount of biomass decreases as *S. obliquus* cells adhere to the photobioreactor wall by aeration instead of collapsing. Accordingly, when the aeration rate was increased to 3 L/min and the light intensity to 150 μmol photons/m^2^s, the amount of lutein reaches to its maximum where the synthesis of lutein was triggered according to the biosynthesis mechanism. Microalgae can be exposed to high light stress in the middle of the day. Because of this excessive accumulation of energy, more ROS are generally created, and they induce the synthesis of antioxidant carotenoids to protect microalgae cells from light damage. The induction mechanism may follow different metabolic pathways depending on the type of light and the strain. For example, cells suddenly exposed to high light intensity can react with induction of the xanthophyll cycle. Similarly, synthesis of lutein, a potent antioxidant, can be induced when exposed to high light. Figure 2a shows a summary of the induction of photoprotection by triggering carotenoid synthesis through high light intensity, and Figure 2b summarizes the overview of lutein biosynthesis in microalgae containing enzymes and corresponding genes involved in biochemical transformation steps [33].

In this study, it was observed that the lipid content of *S. obliquus* increased during saponification process. In fact, it is highlighted that there is a closely related link between lipid and carotenoid production. First, the biosynthesis pathways share the same precursor substrate, acetyl-CoA. Second, the removal of ROS from lipid peroxidation is an important property of carotenoids. Finally, carotenoids are toxic to the cell, so lipid droplets act as “reservoirs” for carotenoids. Direct exposure of microalgae to high light intensity promotes cell growth of microalgae by increasing photosynthesis. Some studies have shown that increased light intensity causes an increase in superoxide and hydrogen peroxide, thus causing oxidative damage to polyunsaturated fatty acids (PUFAs). This resulted in a higher neutral lipid content and lower polar lipid content [34]. It was reported that high light intensity can increase the expression levels of the lycopene beta-cyclase gene, the key enzyme for carotenoid accumulation in microalgae [35]. Lycopene is the precursor to both α-carotene and β-carotene formation after two cyclization reactions. Lycopene β-cyclase (lcy-b) enzyme catalyses the cyclization of both ends of lycopene in order to synthesize β-carotene with two β rings. Meanwhile, the action of lycopene β-cyclase (lcy-b) and lycopene β-cyclase (lcy-e) enzymes catalyses the cyclization of both ends of lycopene to make α-carotene with a β ring and a β ring. This is an important branching point of the carotenoid biosynthesis pathway in microalgae. In this pathway, one branch leads to α-carotene biosynthesis and the other branch to β-carotene biosynthesis. The first is then converted to lutein in two hydroxylation steps, the second to zeaxanthin and then to other carotenoids. Hydroxylation of α-carotene at the C-3 and C-30 positions leads to the formation of lutein and the enzymes involved in these processes are β-carotene hydroxylase and β-carotene hydroxylase, respectively [36]. The stress response mechanisms of different microalgae species can sometimes differ greatly. These systems are very complex. “Omics” approaches such as genomics, transcriptomics, proteomics, metabolomics, and lipidomics offer powerful tools to study the differential expression or regulation of genes under stress conditions [37]. The molecular mechanisms underlying the behaviour of certain species under stress conditions can be made increasingly clear using omics technologies that can guide genetic engineering to improve the biosynthesis of lipids and carotenoids. However, the lack of knowledge about biosynthesis mechanisms makes this subject very interesting and important. The need for more studies in this area is very important in terms of managing the synthesis mechanism.

### 3.3. Preparative chromatography for the isolation of lutein

**Figure 3:**
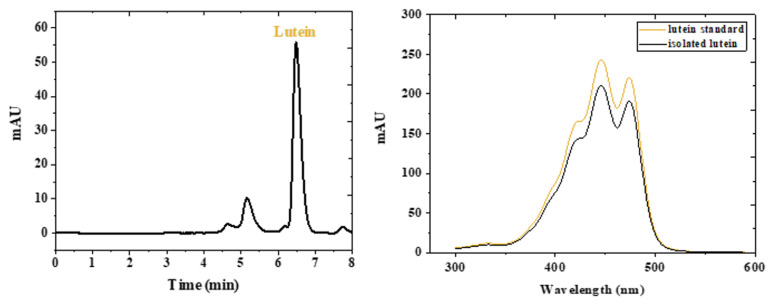
Chromatographic and spectroscopic data for lutein isolated from *S. obliquus*. (a) HPLC chromatogram for *S. obliquus* extract obtained at 446 nm using semiprep C_30_ Carotenoid column, (b) Absorbance spectra of lutein standard and isolated lutein.

In the present study, lutein was isolated with prep HPLC using semiprep C_30_ carotenoid column. The lutein peak was collected between 8.3 min and 9.6 min. Preparative HPLC chromatogram of lutein was depicted in Figure 3a. It was observed that lutein was the second peak in the carotenoid extract of *S. obliquus*, being the major carotenoid peak in this species. In addition, Figure 3b shows the spectroscopic data for the lutein standard and the isolated lutein form *S. obliquus*.

### 3.4. Identification of lutein by mass spectrometry

**Figure 4:**
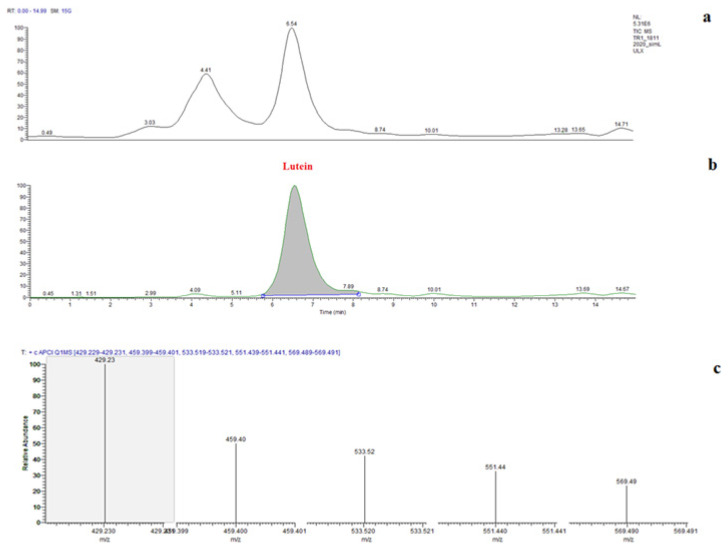
LC-APCI-MS data for lutein from *S. obliquus*. (a) LC chromatogram of *S. obliquus* extract, (b) LC chromatogram of isolated lutein from *S. obliquus* after preparative chromatography, (c) LC-MS/MS spectra of isolated lutein.

The identification was performed according to the following combined information: use of authentic standard, UV-visible spectrum (λ max, spectral fine structure), mass spectra characteristics (protonated molecule ([M+H]^+^) and MS/MS fragments) compared with data available in the literature [38]. For the confirmation of isolated lutein by preparative chromatography, the collected fraction was evaporated and redissolved in 10.0 mL of methanol for LC-MS/MS analysis. LC chromatogram of purified lutein was given in Figure 4a. Lutein was also identified and confirmed by retention time (RT) and selected ion monitoring (SIM) mode. Analysis was performed in positive ion mode and optimized using commercial lutein standard. Figure 4b demonstrates the LC-MS/MS spectra for purified lutein from *S. obliquus*. Monitoring the ions m/z 569.4 [M+H]^+^, 551.4 [M+H-18]^+^, 533.5 [M+H-18-18]^+^, 459.4 [M+H-92-18]^+^ and 429.2 confirms that fucoxanthin was isolated which are consisted with the literature values [2, 39].

## 4. Concluding remarks

Each type of microalgae has different optimal stress conditions for high production of targeted metabolites. Similarly, the stress-based strategy is the most effective way to induce microalgal carotenoid biosynthesis. In this study, induction of lutein synthesis from *Scenedesmus obliquus* was studied by the optimization of light intensity and ventilation rate. Lutein, which was then increased in amount, was easily isolated by preparative chromatography. It has been successfully demonstrated that this strain can be a good resource for industrially producing lutein under the conditions specified by simple and rapid ultrasonic extraction using ethanol. Based on our results, *S. obliquus* can be proposed as an interesting and promising microalga for the commercial production of lutein under the specified conditions given in this work.

## Figures and Tables

**Table 1:** Influence of different aeration rates and light intensities on lutein content and biomass productivity in *S. obliquus*.

Aeration rate and light intensity	Lutein	Specific growth rate	Biomass productivity	Doubling time
(L/min and μmol photon/m^2^s)	(mg/g)	(μ)	(g/L)	(day)
*S. obliquus* 1–50	3.52 ± 0.08	0.38	1.262	1.8
*S. obliquus* 1–150	0.64 ± 0.03	0.49	1.624	1.4
*S. obliquus* 1–300	0.53 ± 0.04	0.42	1.952	1.7
*S. obliquus* 3–50	7.32 ± 0.14	0.28	1.050	2.4
*S. obliquus* 3–150	8.01 ± 0.10	0.30	1.698	2.3
*S. obliquus* 3–300	1.37 ± 0.12	0.35	2.022	2.0
*S. obliquus* 5–50	0.57 ± 0.13	0.31	0.538	2.2
*S. obliquus* 5–150	3.92 ± 0.11	0.43	1.930	1.6
*S. obliquus* 5–300	4.33 ± 0.09	0.33	1.586	2.1
